# Lymphangiome kystique du cou chez un adulte jeune: à propos d'un cas et revue de la littérature

**DOI:** 10.11604/pamj.2020.36.54.21758

**Published:** 2020-06-02

**Authors:** Augustin Kibonge Mukakala, Manix Ilunga Banza, Eric Mbuya Musapudi, Nasser Amisi Lubosha, Trésor Kibangula Kasanga, Dimitri Kanyanda Nafatalewa, Serge Ngoie Yumba, Papy Mukimba Ngabunda, Rodrigue Mupenda Mwenibamba

**Affiliations:** 1 Département de Chirurgie, Cliniques Universitaires de Bukavu, Faculté de Médecine, Université Officielle de Bukavu, République Démocratique du Congo; 2 Département de Chirurgie, Cliniques Universitaires de Lubumbashi, Faculté de Médecine, Université de Lubumbashi, République Démocratique du Congo

**Keywords:** Lymphangiome kystique, tumeur, système lymphatique, Cystic hygroma, tumor, lymphatic system

## Abstract

Les lymphangiomes kystiques sont des malformations congénitales portant sur le système lymphatique. Ce sont des lésions bénignes dysembryoplasiques rares prédominant nettement au niveau de la région de la tête et du cou plus particulièrement dans le triangle cervical postérieur. Ils surviennent généralement durant l'enfance et sont exceptionnels chez l’adulte. Nous rapportons une observation clinique d’un lymphangiome kystique du cou chez un sujet de 22 ans.

## Abstract (en)

Cystic hygromas are congenital malformations affecting the lymphatic system. These are rare benign dyssembryoplastic lesions mainly affecting the head and neck, in particular the posterior triangle of the neck. They usually occur during childhood and exceptionally in adults. We here report a clinical case of cystic hygroma of the neck in a 22-year old subject.

## Introduction

Les lymphangiomes kystiques sont des dysembryoplasies bénignes rares, du système lymphoganglionnaire, responsables d’un syndrome tumoral par prolifération gangiolymphatique [1]. Cette affection signalée pour la première fois en 1828 par Redenbacher et mieux connue depuis les travaux de référence menés par Sabin en 1909 et 1912 [1]. Leur localisation anatomique est presque exclusivement cervico-faciale [2] et leur révélation clinique est généralement très précoce [1]. Le lymphangiome est une malformation congénitale bénigne des vaisseaux lymphatiques vu presque exclusivement chez l’enfant de moins de deux ans. Il est extrêmement rare chez l’adulte. Ces malformations sont souvent retrouvées au niveau de la tête et du cou. Dans la cavité buccale, la langue est le site le plus fréquent du lymphangiome, cependant cette lésion est exceptionnellement rapportée au niveau du plancher buccal [3]. Il est considéré comme une séquestration du tissu lymphatique qui a conservé son potentiel de croissance. Trois variantes ont été décrites pour ces lymphangiomes [4] à savoir le lymphangiome capillaire, caverneux et kystique. L’observation que nous rapportons est une variété kystique diagnostiquée tardivement chez un patient jeune adulte de 22 ans, avec comme objectif principal de partager notre expérience sur ce cas très rare à cet âge.

## Patient et observation

**Figure 1:**
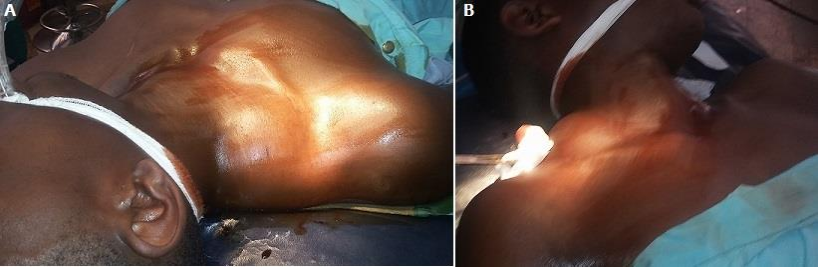
La masse siège sur la partie inféro-latérale droite à la base du cou (A,B)

Un sujet de 22 ans sans antécédents particulier consulte au *Skyborne Hospital* de Bukavu pour une tuméfaction au cou apparue 4 ans avant, et était de petite taille au début, mais qui au fil du temps aurait augmenté de volume progressivement jusqu’à atteindre la taille d’une mangue sans notion de traumatisme. A l’examen physique, la masse siège sur la partie inféro-latérale droite à la base du cou (Figure 1) mesurant 13cm de grand axe longitudinal et 7cm de petit axe transversal occupant la moitie interne du creux sus-claviculaire droite. La masse était indolore, régulière, rénitente, mobilisable et non pulsatile. Il n’avait pas de troubles sensitivomoteurs au membre supérieur homolatéral et les pouls radial et cubital étaient perceptibles. Une échographie avait été faite et avait montré une masse à contenu anéchogène, multicloisonée. La radiographie standard du cou faite, avait montré une opacité arrondie basi-cervicale droite à limite externe régulière, sans signe de compression des organes du voisinage ni image de calcification, avec un contour interne flou.

Sur base de ces éléments, un diagnostic de masse kystique du cou était évoqué et une intervention chirurgicale avait été indiquée. En peropératoire, on retrouve une masse à contenu, liquidienne entre la veine jugulaire interne droite et les fibres du sterno-cléido-mastoïdien droit sans aucune autre malformation des vaisseaux. L’exérèse de la masse s’était faite sans incident et les suites opératoires étaient simples. La masse avait été envoyée au laboratoire de l’Hôpital Provincial Général de Référence de Bukavu pour un examen anatomo-pathologique, qui avait conclu à un lymphangiome kystique dont les parois étaient faites de tissu fibromusculaire avec beaucoup de follicules lymphoïdes à centre, clairs et les canaux lymphatiques étaient dilatés avec un contenu mélangé de la lymphe et du sang.

## Discussion

Le lymphangiome kystique ou *hygroma kystica* est une tumeur bénigne rare. Il peut se localiser dans l’abdomen, le thorax, et au niveau du cou. La localisation cervicale se rencontre plus dans l’enfance: 90% avant l’âge de 20 ans, mais peut être découverte a tout âge de vie en raison de la latence d’évolution [5-7], comme cela a était le cas de notre patient âgé de 22 ans. D’autres localisations ont été citées dans la littérature: la localisation splénique, colique, rétropéritonéale [8, 9], rétropéritonéale [10] et la localisation funiculaire (au niveau du cordon spermatique) [11]. L’observation de KARIM fait état d’une localisation cervico-thoracique [12]. Pour notre observation, le siège cervical serait justifié par l’hypothèse selon laquelle, elle résulterait de la migration d’éléments lymphatiques initialement séquestrés à l’étage cervical [13]

Deux théories pathogéniques sont évoquées dans la littérature: la théorie traumatique explique la survenue de ces Kystes par une obstruction ou une contusion lymphatique, mais cette théorie est rarement confirmée par l’histoire clinique. [3] et La théorie congénitale qui est la plus admise actuellement. Le lymphangiome proviendrait d’une séquestration de sac lymphatique embryonnaire qui se remplirait progressivement de liquide lymphatique [11]. L'échec de l'établissement d’anastomose entre les vaisseaux normaux et pathologiques et l’accumulation de liquide lymphatique seraient responsables de la genèse de cette lésion [8].

Pour notre observation, le lymphangiome était de localisation cervicale chez un adulte jeune et nous pensons que cette deuxième théorie pathogénique expliquerait mieux notre cas étant donné que chez notre patient, la masse avait évolué progressivement au fil des années. Le siège de lymphangiome kystique est ubiquitaire, et leur localisation préférentielle est le triangle cervical postérieur, avec extension médiatisnale dans 10% des cas. Pour Minocha etal. [14], ces tumeurs sont fréquemment localisées au niveau de la tête, du cou, dans l’aisselle et en intra-abdominale. L’observation d’Eric Mbuya etal. [13] rapporte que, le siège du lymphangiome était mammaire et plusieurs auteurs ont rapporté ce siège dans la littérature [4, 14-18]. Elboukhari Ali etal. [3] avaient rapporté un cas de lymphangiome du plancher buccal étendu à la région sous mandibulaire.

**Figure 2:**
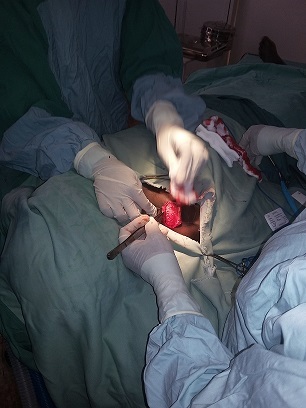
tracé de l’incision suivi de l’exérèse

**Figure 3:**
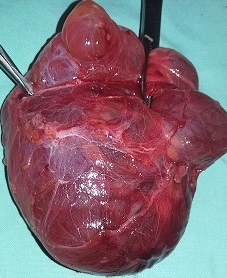
Masse kystique

Trois types de lymphangiomes peuvent être distingués; les lymphangiomes capillaires comprenant des petits vaisseaux a lumière étroite, les lymphangiomes caverneux à lumière dilatée, anfractueuse et inter-communicante et le lymphangiomes kystiques ou hygroma kystique présentant de larges cavités confluentes remplies de liquide jaune clair. [11]. La symptomatologie du lymphangiome kystique est fonction de la taille de la tumeur et de la topographie de la masse. Très souvent il est révélé par une tuméfaction cervicale en dehors de tout traumatisme [19], comme c’était le cas pour notre patient. Dans notre contexte, l’échographie pose bien le diagnostic d’un lymphangiome kystique en montrant une masse à contenu anéchogène, multicloisonée avec un renforcement postérieur et la radiographie standard du cou a un intérêt dans la recherche d’une déviation trachéale et/ou d’un prolongement médiatisnal de la masse. Le traitement du lymphangiome kystique du cou est essentiellement chirurgical et le diagnostic de certitude se fait par l’examen anatomopathologique [7, 11]. Pour notre cas, nous avons pratique une cervicotomie latérale droite qui nous avait permis d’avoir un bon plan de clivage vu le volume de la masse et ainsi obtenir une exérèse totale de la masse kystique (Figure 2, Figure 3). L’évolution post-opératoire était simple et le patient n’avait pas présenté une récidive, vu 6 mois, un an et deux ans après.

Trois critères histologiques caractérisent les lymphangiomes kystiques: une formation kystique, la présence des cloisons à stroma conjonctif pourvu de tissu lymphoïde et de muscle lisse, élément capital pour le diagnostic et un revêtement endothélial tapissant ces formations kystiques la tumeur a une origine vasculaire [20]. En ce qui concerne notre cas, l’examen anatomo-pathologique avait conclu à un lymphangiome kystique dont les parois étaient faites de tissu fibromusculaire avec beaucoup de follicules lymphoïdes à centre clairs, et les canaux lymphatiques étaient dilatés avec un contenu mélangé de la lymphe et du sang.

Nous avons retrouvé plusieurs classifications dans la littérature. Certains auteurs préfèrent classer les lymphangiomes en lymphangiomes capillaires, caverneux et kystiques; mais actuellement il est préférable de les diviser en lésions microkystiques, macrokystiques et mixte [17]. Pour notre observation, l’examen histopathologique avait classé notre masse comme étant un lymphangiome macrokystiqe étant donné qu’il était composé d’espaces kystiques supérieurs à 2cm^3^. D’autres moyens thérapeutiques ont été rapportés dans la littérature mais leur efficacité n’a jamais été démontrée. C’est le cas de la radiothérapie, le drainage sous scopie ou écho-guidée, et l’injection intraveineuse de la cyclophosphamide pour obtenir une sclérose chimique [19] .Pour notre cas, une cervicotomie latéro-basale droite nous avait permis de faire l’exérèse de la masse sans difficulté et les suites opératoires avaient été simples.

## Conclusion

Le lymphangiome kystique du cou est une tumeur bénigne qui est connue habituellement par sa survenue à un âge très précoce et son évolution lente. Sa découverte à l’âge adulte est exceptionnelle. Le constat clinique couplé à l’imagerie permettent d’éliminer les autres masses cervicales et suffisent pour penser au lymphangiome kystique du cou. Une exérèse complète et prudente reste le seul traitement et facilite la confirmation diagnostique par l’examen histopathologique.

## Conflits d’intérêts

Les auteurs ne déclarent aucun conflit d'intérêts.
